# Oxygen-Releasing Antibacterial Nanofibrous Scaffolds for Tissue Engineering Applications

**DOI:** 10.3390/polym12061233

**Published:** 2020-05-29

**Authors:** Turdimuhammad Abdullah, Kalamegam Gauthaman, Ahmed H. Hammad, Kasturi Joshi Navare, Ahmed A. Alshahrie, Sidi A. Bencherif, Ali Tamayol, Adnan Memic

**Affiliations:** 1 Center of Nanotechnology, King Abdulaziz University, Jeddah 21589, Saudi Arabia; 1202908@gmail.com (T.A.); ahhhassan@kau.edu.sa (A.H.H.); aalshahri@kau.edu.sa (A.A.A.); 2 Center of Excellence in Genomic Medicine Research, King Abdulaziz University, Jeddah 21589, Saudi Arabia; Kgauthaman@kau.edu.sa; 3 Faculty of Medicine, AIMST University, Semeling, Bedong, Kedah 08100, Malaysia; 4 Electron Microscope and Thin Films Department, Physics Division, National Research Centre, Dokki, Giza 12622, Egypt; 5 Department of Chemical Engineering, Northeastern University, Boston, MA 02115, USA; k.joshinavare@northeastern.edu; 6 Department of Physics, Faculty of Science, King Abdulaziz University, Jeddah 21589, Saudi Arabia; 7 Department of Bioengineering, Northeastern University, Boston, MA 02115, USA; s.bencherif@northeastern.edu; 8 Harvard John A. Paulson School of Engineering and Applied Sciences, Harvard University, Cambridge, MA 02138, USA; 9 UMR CNRS 7338 Biomechanics and Bioengineering, University of Technology of Compiègne, Sorbonne University, 60200 Compiègne, France; 10 Department of Biomedical Engineering, University of Connecticut, Farmington, CT 06030, USA; atamayol@uchc.edu

**Keywords:** oxygen-releasing scaffold, PGS/PCL, calcium peroxide, electrospinning, biodegradability, antibacterial properties, biocompatibility

## Abstract

Lack of suitable auto/allografts has been delaying surgical interventions for the treatment of numerous disorders and has also caused a serious threat to public health. Tissue engineering could be one of the best alternatives to solve this issue. However, deficiency of oxygen supply in the wounded and implanted engineered tissues, caused by circulatory problems and insufficient angiogenesis, has been a rate-limiting step in translation of tissue-engineered grafts. To address this issue, we designed oxygen-releasing electrospun composite scaffolds, based on a previously developed hybrid polymeric matrix composed of poly(glycerol sebacate) (PGS) and poly(*ε*-caprolactone) (PCL). By performing ball-milling, we were able to embed a large percent of calcium peroxide (CP) nanoparticles into the PGS/PCL nanofibers able to generate oxygen. The composite scaffold exhibited a smooth fiber structure, while providing sustainable oxygen release for several days to a week, and significantly improved cell metabolic activity due to alleviation of hypoxic environment around primary bone-marrow-derived mesenchymal stem cells (BM-MSCs). Moreover, the composite scaffolds also showed good antibacterial performance. In conjunction to other improved features, such as degradation behavior, the developed scaffolds are promising biomaterials for various tissue-engineering and wound-healing applications.

## 1. Introduction

Organ failure and tissue defects are one of the most decisive threats to human life, and the number of patients in need of tissue and organ transplants has been on the rise [1]. However, there is a significant mismatch between the available donors and the clinical need. In the US alone, approximately 116,000 patients are in queue for organ transplantation, and postponement of the transplantation surgery could be responsible for as many as 20 deaths every day [1,2]. Tissue engineering has emerged as a way to address these shortcomings, in which tissue-like structures are used as grafts for implantation [3]. Upon implantation, the engineered tissue is meant to provide the required physical support and stimulate regeneration of tissues, leading to their functional recovery [4,5,6].

Although tissue engineering has shown great potential in vitro, its clinical success has been limited to a few tissues only [7,8]. One of the critical limiting steps in this process is oxygen supply deficiency of engineered tissues, which often occurs due to improper or insufficient angiogenesis and vascularization [7,8]. An adequate supply of oxygen is critical for cell viability and growth, in order to facilitate tissue regeneration. Lack of oxygen resources in the transplanted engineered tissues could cause hypoxia, which results in cell apoptosis and tissue necrosis [9,10]. Neoangiogenesis, a vital process in tissue engineering, can promote new blood vessel formation and infiltration throughout the scaffold [11]. However, it often takes one-to-two weeks for the host vasculature to infiltrate the inner layers of transplanted scaffolds [12,13]. This process might take longer in patients suffering from underlying conditions, such as diabetes [14]. Similarly, oxygen supply within the scaffold at the wound area prior to the neovascularization would be critical to maintain adequate cell metabolism and tissue growth [7,8]. Therefore, developing long-lasting oxygen-releasing scaffolds could potentially overcome such oxygen supply shortages for both tissue-engineering applications [8,12].

Tissue-engineered scaffolds can be fabricated by numerous techniques, and electrospinning has become increasingly popular [3]. In general, electrospinning is a versatile, cost-effective, scalable and robust technique to produce nanofibrous scaffolds, which resemble the native extracellular matrix (ECM) of human tissues [15]. This technique provides a tremendous opportunity in scaffold design, in which a large variety of materials, such as polymers, ceramics and inorganic materials, can be easily combined to form a nanofiber composites with unique and multifunctional features [15,16,17,18]. Furthermore, loading and releasing bioactive agents can be controlled by multiple strategies, such as initial blending, controlling polymer hydrophobicity, nozzle configuration and physical/chemical conjugation [19,20,21]. Finally, it is a one-step approach to regulate shape, dimension and structure of scaffolds by tuning electrospinning parameters (such as voltage, concentration, feed rate and traveling distance of fibers) and selection of collector pattern [22]. Based on this strategy, the customized scaffold can be fabricated to fulfill required conditions for a specific organ.

Solid peroxide particles, including magnesium peroxide, calcium peroxide (CP) and sodium percarbonate, are some of the most suitable sources to produce oxygen within scaffolds, as they are relatively stable against decomposition during the electrospinning process [23,24,25]. Meanwhile, at tuned concentration, they can be utilized for tissue-engineering applications due to their low toxicity [23,26]. Once they are exposed to an aqueous environment, they react with water and release oxygen (Equations (1) and (2)) [7,27]. Among them, CP is preferentially used as oxygen-releasing agent, because of its low cost and wide commercial availability [7,28]. Additionally, a number of studies suggested that CP exhibits good antimicrobial properties, which could also play an important role for not only tissue engineering but also wound-healing and -dressing application [29]. Oxygen generation due to the chemical decomposition of CP is in two stages, as shown below, in which hydrogen peroxide is unstable and quickly breaks into water and oxygen [7]. Depletion of hydrogen peroxide could be facilitated with catalase, an enzyme found in nearly all living organisms [7].

CaO_2_ + 2H_2_O → Ca(OH)_2_ + H_2_O_2_ (1)

2 H_2_O_2_→ O_2_ + H_2_O (2)

**Figure 1:**
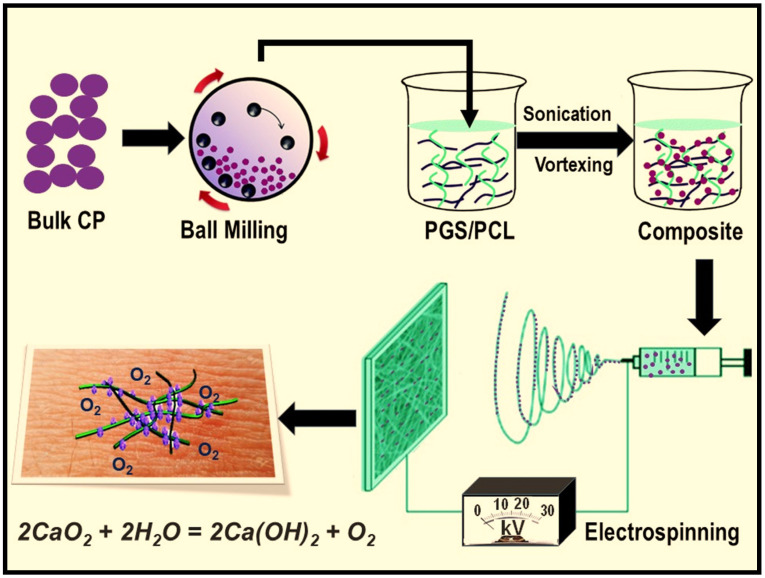
Schematic illustration describing the fabrication process of oxygen-releasing scaffolds by electrospinning.

Poly(glycerol sebacate) (PGS) is a biocompatible elastomeric polyester which has superior surface degradation, faster degradation rate and lower inflammatory responses as compared to other synthetic biopolymers [30,31,32]. However, electrospinning of PGS alone is challenging, and it needs to be performed with a carrier polymer [30,33,34]. On the other hand, poly(*ε*-caprolactone) (PCL) is an FDA-approved, biocompatible and flexible aliphatic polyester that can be easily electrospun to form uniform fibrous structure. Additionally, PCL undergoes a slow degradation process, and once combined with PGS the degradation rate can be tuned by adjusting their ratio in the blend. We previously designed different types of electrospun PGS/PCL nanofibrous platforms [30,33,34] and demonstrated their potential in wound dressing [35], electronic medical device [33,36] and on-demand drug-delivery application [32]. In the current study, we fabricated oxygen-releasing PGS/PCL composite scaffolds by incorporating ball-milled CP nanoparticles into the polymer matrix during electrospinning (Figure 1). First, we characterized the surface morphology, structural integrity and thermal behavior of the composite fibrous scaffolds. Next, we evaluated their biodegradation rate and oxygen-release kinetics (i.e., measurement of dissolved oxygen (DO) concentrations). We also tested the antimicrobial activity of the composite scaffolds against *Staphylococcus aureus* (*S. aureus*), a major bacterial human pathogen. Finally, the effect of oxygen release from the scaffolds on cell metabolic activity was investigated, using primary bone-marrow-derived mesenchymal stem cells (BM-MSCs) as a model cell line.

## 2. Materials and Methods

### 2.1. Materials

PCL (*M*_w_ = 80,000), glycerol, sebacic acid, CP (200 mesh size or 74 μm, 75% purity), catalase (from bovine serum, 5000 unit/mg), sodium dodecyl sulfate (SDS), chloroform and ethanol were purchased from Sigma-Aldrich (St. Louis, MO, USA). Phosphate-buffered saline (PBS) and fetal bovine serum (FBS) were both purchased from Thermo Fisher Scientific Inc. (Fair Lawn, NJ, USA). Dulbecco’s Modified Eagle’s Medium (DMEM, 1X) was obtained from Lonza, USA. PGS (*M*_w_ = 12,000) was synthesized according to a previously reported procedure [16]. Briefly, polycondensation of mixed glycerol and sebacic acid with 1:1 molar ratio was performed at 120 °C, under approximately 5 Pa of high vacuum condition. CP was ball-milled in a horizontal oscillatory mill (Retsch, PM 400, Haan, Germany), operating at 25 Hz for 24 h, to produce ultrafine particles prior to electrospinning. A total of 250 mL of steel cells and steel balls of 1.5 cm in diameter (weight ratio between balls and CP is 10:1) were used as a milling agent.

### 2.2. Electrospinning

PGS (10% *wt./v*) and PCL (10% *wt./v*) were dissolved in mixture of chloroform and ethanol at 9:1 ratio. Then 1%, 2.5%, 5% and 10% (*wt./v*) of CP nanoparticles were added into the solution under vigorous stirring, followed by 1 h sonication and 2–5 min vortex mixing, to obtain a homogeneous mixture. Electrospinning was conducted in a Nanon 101A electrospinning setup (NANON Supply, MECC, Fukuoka, Japan) at 23 kV of applied voltage, 15 cm of distance and 1.2 mL/h of feed rate. An 18 G needle was used for electrospinning, and the needle head was cleaned every 2 min, to prevent needle blockage. A flat aluminum sheet was used to collect the generated fibers. Catalase (1 mg/mL) was incorporated into the select solutions for the oxygen-release and biocompatibility studies. The abbreviated scaffold names and their initial mixing compositions can be found in Table 1.

### 2.3. Characterization

Surface micrograph of the prepared scaffolds were obtained by using a field emission scanning electron microscopy (FESEM, JEOL JSM 7600F, Tokyo, Japan). The elemental analysis of the composite scaffolds was acquired by using energy-dispersive X-ray spectroscopy (EDX) attached to the FESEM. The fiber size distribution was determined according to a custom-designed image-processing algorithm developed in Matlab (R2016a, MathWorks, Natick, MA, USA), as described by our group previously [22]. The size distribution of the ball-milled CP nanoparticles was calculated by direct measuring edges of 200 different particles in “Image J” software (1.50i, National Institutes of Health, Bethesda, MD, USA). Moreover, atomic resolution transmission electron microscopy (JEOL JSM-200F, Tokyo, Japan) was used to image the micro/nanostructural features of the scaffolds on uncoated carbon grid.

The X-ray diffraction (XRD) patterns of the scaffolds and CP nanoparticles were recorded, using ULTIMA IV XRD systems (Rigaku, Japan) attached with Cu Ka radiations. The thermal-transition characteristics of the scaffolds were analyzed with a differential scanning calorimeter (DSC-60, Shimadzu Corporation, Japan), across the temperature range of 25 to 150 °C.

The protein-adsorption capacity of the scaffolds was determined by soaking the scaffolds in 10% of FBS for 24 h, prior to extracting the adsorbed protein from the scaffold by using SDS. A nanodrop 2000 system (Thermo Fisher Scientific, Fair Lawn, NJ, USA) was used to calculate the amount of adsorbed protein, as described by others [16].

### 2.4. Degradation and Oxygen Release

Degradation was analyzed based on mass loss of the scaffolds over time. Scaffolds (~15 mg) were immersed into PBS and incubated at 37 °C. The weight was calculated as (*Wt/Wo*) × 100%, where *Wo* was the initial sample weight and *Wt* was the sample weight at time, t. The released oxygen from the scaffolds were determined according to the DO in deionized water (DIW). Circular scaffolds with a diameter of 2 cm were inserted into a 6-well cell-culture plate containing 10 mL of DIW. The amount of DO was measured by Professional Digital Large LCD Dissolved Oxygen Meter Water Quality Tester with ATC 99 Memory Function (Gain Express Holdings Ltd., Hong Kong, China). The data were recorded for a week and normalized for the initial sample weight in the DIW. The concentration of DO in DIW was measured and normalized based on the following equation:

DO (mg/g scaffold) = *V*_x_(DO_M_-DO_R_)/*W*_s_ (3)

where DO_M_ is the concentration of DO in the scaffold-contained DIW (unit is mg/L), DO_R_ is concentration of DO in pure DIW (unit is mg/L), *V*_x_ is volume of DIW (unit is L) and *W*_s_ is the weight of the inserted scaffold (unit is g).

### 2.5. Antibacterial Performance and Metabolic Activity

Disk diffusion tests were used to examine the antimicrobial performance of scaffolds. Secluded colonies of *S. aureus* were inserted into Lenox broth (LB) media containing 100 μg/mL ampicillin (Sigma-Aldrich, St. Louis, MO, USA) and cultured overnight at 37 °C. Then, 200 μL of media were evenly distributed on the top of LB-agar plate containing 100 μg/mL of ampicillin (Sigma-Aldrich, St. Louis, MO, USA). Finally, disks punched from an electrospun sheet were attached on the surface of bacteria strain and incubated at 37 °C for 18 h.

To evaluate the metabolic activity of cells, the scaffolds were collected on 15 mm of glycol-modified polyethylene terephthalate (PETG) rings and inserted into a 24-well culture plate. The rings were manufactured by 3D printing (Ender-3, Creality3D, Shenzhen, China), and they were also inserted into the culture plate, as a control. The scaffolds were sterilized under UV for 1 h, followed by washing with ethanol 4 or 5 times. Patient-derived bone-marrow-derived mesenchymal stem cells (BM-MSCs) (2 × 10^4^ cells/well) were cultured on the scaffolds, at standard culture conditions of 37 °C, in a 5% CO_2_ incubator, with regular media change every 48 h. An MTT reagent kit (3-(4,5-dimethylthiazolyl-2)-2,5-diphenyltetrazolium bromide (Sigma-Aldrich, St. Louis, MO, USA) was used to assess cell viability. The culture media was replaced with 100 μL of fresh media, and 10 μL of MTT reagent was added, followed by incubating the cells for 4 h. The resulted formazan products were dissolved, using 100 μL of the detergent and incubated for another 2 h in the dark. A microplate reader (SpectraMax i3, San Jose, CA, USA) was used to spectrophotometrically measure absorbance at 570 nm the wavelength, while 630 nm wavelength was used as the reference. The cell metabolic activity (MA) of the scaffolds were presented as percentage, according to optical density ratio between the scaffold and control, using the following equation:

MA (%) = OD_S_·100/OD_C_ (4)

where OD_S_ is MTT optical density value for the scaffold, and OD_C_ is average MTT optical density value for the control (*n* = 3).

### 2.6. Statistical Analysis

All of the experiments were repeated three times. The data are shown as mean ± standard deviation (SD). The data were analyzed by using origin software (OriginPro 8.0, Origin Lab Inc., Northampton, MA, USA).

## 3. Results

As presented in Figure 1, CP nanoparticles were prepared first by using high-energy ball-milling, as previously described [37], prior to being mixed in a PGS/PCL solution for electrospinning. Since CP is a heavy compound (density ~2.91 g/cm^3^), the polymer was first dissolved, and then CP nanoparticles were added into the viscous solution. The uniform dispersion of CP nanoparticles in the solution was further achieved, using ultra sonication and vigorous vortex mixing. In our previous studies, we applied a voltage of 19.5 kV, 0.9 mL/h of feed rate and a 27 G needle to prepare electrospun PGS/PCL scaffolds [33]. Whereas, here, we applied a higher voltage and a faster feed rate, and used a larger 18 G needle, in order to control fiber uniformity and avoid particle aggregation in the syringe or needle during the electrospinning process.

**Figure 2:**
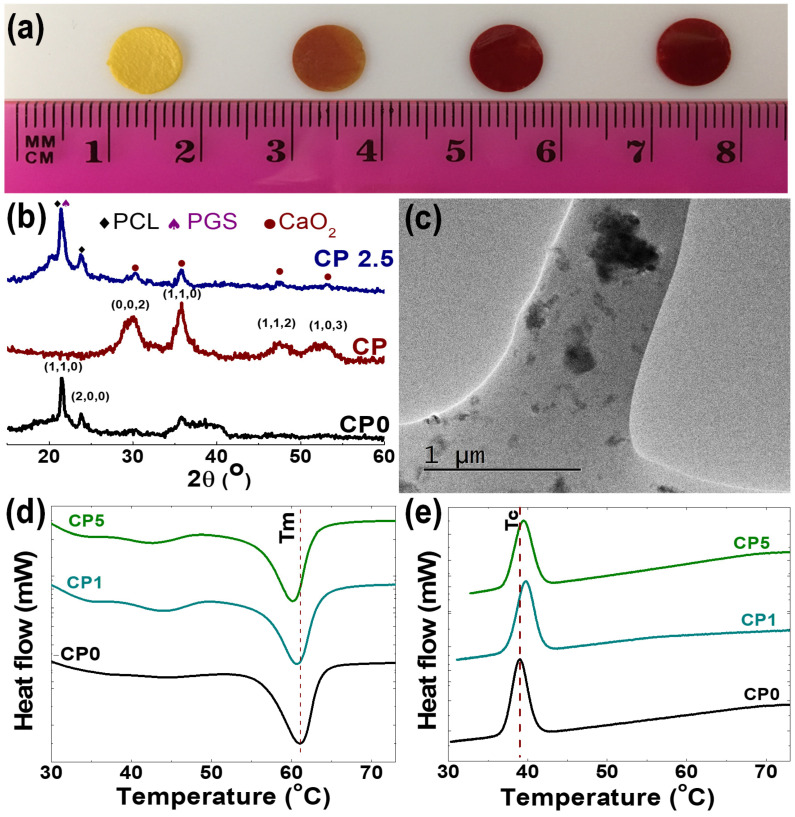
(**a**) Alizarin red S staining of the electrospun sheet with different concentration of CP, confirms incorporation of CP in PGS/PCL polymer network. (**b**) XRD spectra of CP nanoparticles, PCL/PGS scaffold and composite scaffold. (**c**) TEM image of the electrospun composite nanofiber demonstrates embedding of CP nanoparticles within the fiber. (**d**,**e**) DSC curve of PGS/PCL scaffold with different concentration of CP scaffold during heating (**d**) and cooling (**e**).

The presence of CP in the composite scaffolds was demonstrated by Alizarin red S staining, which is usually used for identification of inorganic calcium (Figure 2a). The composite scaffolds showed a clear red staining, and staining intensity was proportional to the concentration of CP embedded within the scaffolds. We also confirmed CP incorporation at various concentrations in PGS/PCL electrospun during the electrospinning by EDX elemental mapping (Table 2). All the elements were detected by EDX except for hydrogen, since it does not have valance independent K-shells [38]. Similarly, the XRD pattern of the composite scaffolds showed preserved crystalline structure of both PCL/PGS polymer blends and CP nanoparticles (Figure 2b). For the PCL/PGS blends, peaks appeared at 21° and 23.5°, mainly attributed to XRD spectra of PCL [39,40]. This is supported by the literature, as others have shown that PGS has a broad peak near to 21° [40]. In the case of the CP nanoparticles, peaks can be observed at 30.3°, 35.8°, 47.5°, 53.3° and 60.7°, which are index of (002), (110), (112) and (103) reflection of CP, respectively [41]. All of the peaks could be found in the composite scaffolds without any significant shifts, and peak positions are consistent with their standard Joint Committee on Powder Diffraction Standards (JCPDS) cards [40,41]. TEM image of an electrospun composite fiber is presented in Figure 2c, in which numerous dark spots can be observed, confirming that CP nanoparticles were successfully embedded within the fibers during electrospinning. Next, we examined the phase-transition behavior of the scaffolds during heating and cooling (Figure 2d,e). The CP-free PGS/PCL scaffolds showed a melting temperature of a 61.5 °C and a crystallization temperature of 39 °C, which are typically attributed to thermal behavior of PCL in the scaffold [42,43]. When the CP concentration was increased, the melting temperature decreased, while the crystallization temperature increased for the composite scaffolds. This set of data suggests that CP nanoparticles may be acting as a nucleation agent in the PCL/PGS polymer matrix [44,45].

**Figure 3:**
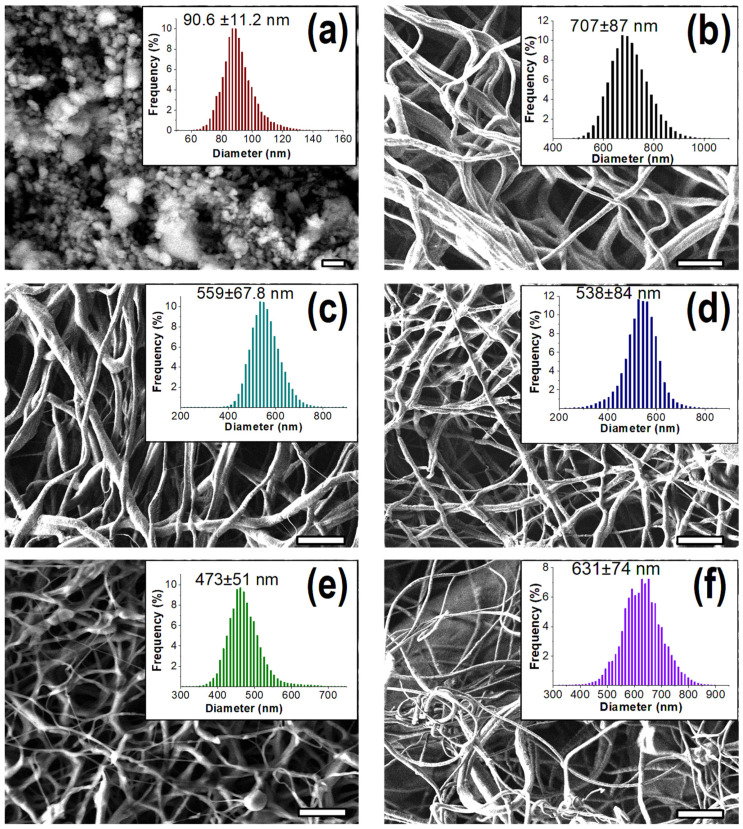
SEM images and size distributions of ball-milled CP particles (**a**), PCL/PGS scaffold without CP (**b**) and PCL/PGS scaffold with 1% (**c**), 2.5% (**d**), 5% (**e**) and 10% (**f**) CP. Scale bars = 1 µm (**a**) and 10 µm (**b**–**f**).

SEM micrographs and size distribution of CP-embedded composite fibers depicting their morphological features are shown in Figure 3. Here, we utilized a customized image processing technique developed in Matlab software, to determine the fiber size. This technique provides a statistically reliable, normal/Gaussian distribution curve with finical density approximation [22]. The original particle size of CP was about 74 μm that was reduced to 90.6 ± 11.2 nm, following high-energy ball-milling. CP-free PGS/PCL scaffold showed a smooth and uniform fibrous structure with a 707 ± 87 nm diameter. The smoothness and uniformity of the scaffolds were not altered within addition of CP nanoparticles up to 5%. Moreover, scaffold fiber diameters significantly decreased with increased CP percentage, and 473 ± 51 nm was the lowest fiber size achieved for the composite scaffold with 5% of CP. Nevertheless, 5% or higher CP content initiated bead formation within fibers. This could be due to the aggregation effect of CP nanoparticles in the solution during the process of electrospinning.

**Figure 4:**
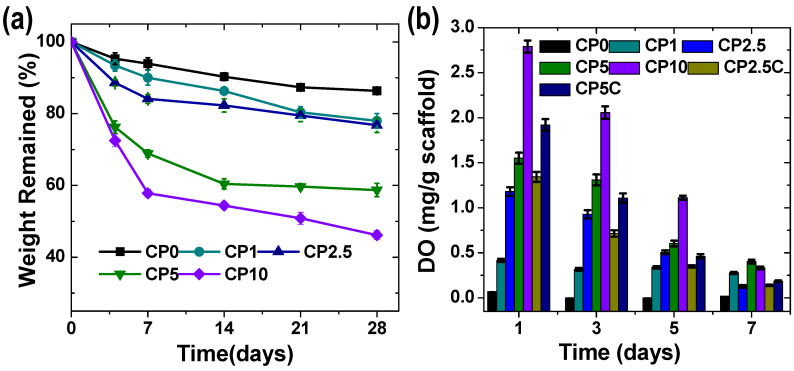
(**a**) Degradation profiles in PBS buffer, and (**b**) oxygen-releasing kinetics of the composite PGS/PCL scaffolds at various CP concentrations.

Next, we analyzed the degradation behavior of the engineered scaffolds by calculating weight change over time through immersion of the samples into PBS buffer. Typically, the composite scaffolds showed a faster degradation rate when compared to CP-free PGS/PCL scaffolds, which had ~14% mass loss in PBS over 4 weeks (Figure 4a). The composite scaffolds underwent a two-stage degradation profile, in which a quick degradation occurred in the first week, followed by a slow degradation. Meanwhile, the degradation rate was highly proportional to the percentage of CP in the scaffold. For example, CP10 scaffolds exhibited 42 ± 1% of mass loss in the first week, and approximately 11% additional mass loss occurred within the next three weeks. We performed elemental mapping for the composite scaffolds, following degradation, and found that the scaffolds were calcium free (Supplementary Figure S1). Meanwhile, we also evaluated the protein-adsorption capacity of the scaffolds and noticed that the incorporation of CP actually enhanced protein adsorption (Supplementary Figure S2).

Next, in order to measure oxygen-release kinetics, the scaffolds were soaked in deionized water, and the concentration of DO was monitored over time (Figure 4b). Overall, all composite scaffolds exhibited their highest oxygen release at day one, and a gradual decrease of oxygen occurred afterward. The scaffold with 10% CP initially showed the highest oxygen release, whereas a more sustainable release occurred for the scaffold with 5% CP. These results suggest that better fiber morphology of electrospun scaffold could provide a stronger interaction between the polymer and particles and improve oxygen-release kinetics of CP. We also introduced catalase into the scaffold, to enhance the rate of oxygen release, which showed higher initial burst release of oxygen. It was expected that the incorporation of catalase would quickly deplete hydrogen peroxide, a by-product of CP decomposition, known to be potentially toxic to human cells and tissues [46].

**Figure 5:**
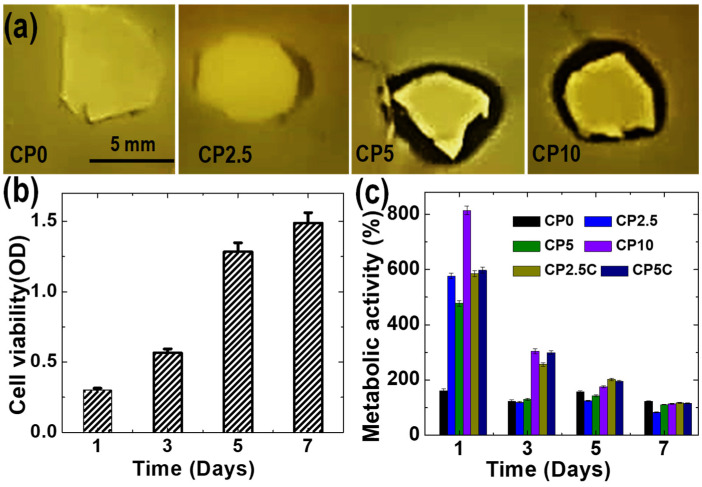
(**a**) Antibacterial activity of the scaffolds against *S. aureus* presented by zone of inhibition. (**b**) Cell viability results for the scaffold-free 3D-printed ring (control), presented by the optical density (OD) value for the MTT assays. (**c**) Cell metabolic activity of BM-MSCs in the scaffold. The data were normalized according to Equation (4) and represented as mean ± SD (*n* = 3).

We also tested the antibacterial performance of the composite scaffolds against *S. aureus*. While CP-free and CP2.5 scaffolds did not show any clear inhibition zone, CP5 and CP10 scaffolds exhibited a clear inhibition zone (Figure 5a). The antibacterial activity at high CP concentrations is likely due to residual hydrogen peroxide, a by-product of CP decomposition in aqueous solvents, as well as calcium hydroxide, which has also shown to be antibacterial [47]. Next, we evaluated the influence of oxygen release from the composite scaffolds on the metabolic activity of patient-derived BM-MSCs in vitro. We applied MTT assay to analyze the metabolic activity of cells across the different scaffolds, and the results where compared to a scaffold-free group (control). Within a week of incubation, an increased cell viability was observed for the control group, suggesting that the printed rings are cytocompatible (Figure 5b). On the first day of cell culture, the metabolic activity of cells on the composite scaffolds was much higher than the control, and it was proportional to the CP concentration (Figure 5c). The difference for the metabolic activity of cells between the control and the composite scaffolds decreased for longer incubation time. Furthermore, a similar metabolic activity for the cells was observed for both composite scaffolds and control at day seven. It is also worth highlighting that the addition of catalase improved the metabolic activity of cells on the composite scaffolds, most likely due to hydrogen peroxide depletion [46].

## 4. Discussion

Supplying oxygen in a controlled and sustained fashion is a critical consideration for translating tissue-engineered scaffolds for clinical applications [7]. One promising strategy is to incorporate oxygen-releasing nanoparticles. Specifically, CP, along with MgO_2_ and Na_2_CO_3_,is an example of a material that could be used to release oxygen sustainably [28]. To control the oxygen-release kinetics of these nanomaterials, hydrophobicity and degradability of the surrounding polymers are important factors to consider [25]. For example, super-hydrophilic polymers or polymers that quickly degrade usually are not recommended for the encapsulation of solid peroxide nanoparticles, since they lead to fast oxygen depletion [26]. Reciprocally, oxygen-release rate could be too slow, if highly hydrophobic polymers are used. Here, we selected PGS/PCL blend for entrapping CP, which shows a sustainable degradation rate and moderate hydrophilicity. Moreover chloroform-ethanol solvent had no adverse effects on the stability of CP during mixing prior to electrospinning [48].

Electrospinning of colloidal suspension is often challenging, due to the aggregation of solid microparticles [49,50]. In our studies, we effectively avoided CP accumulation during the solution preparation and electrospinning by previously using a high-energy ball-milling technique to reduce the size of CP. Therefore, we were able to embed CP particles up to 5% into the electrospun fibers, without affecting the uniformity of the formed fibrous mesh. However, the fiber size was significantly reduced. Furthermore, the physiochemical integrity and crystalline structure of CP nanoparticles remained unchanged during the electrospinning process, and the scaffolds were room-temperature stable.

Matching scaffold degradation kinetics to native tissues is another key design criteria for developing tissue-engineering scaffolds [12,51]. Optimally, as scaffold degradation occurs, native ECM starts to replace the scaffold, to facilitate tissue growth and repair [52,53]. Our results showed that the degradation rate of the scaffolds can be tuned by adjusting the concentration of individual components. For instance, the amount of incorporated CP greatly influenced the degradation rate of the composite scaffolds. This finding could be attributed to the separation of CP nanoparticles from the scaffold under an aqueous condition, to generate oxygen, which facilitates faster degradation. Additionally, the protein-adsorption results suggest that the release of CP from the scaffolds could also increase surface area and porosity of the scaffold, which could also contribute to faster degradation.

Another important consideration is inhibiting microbial contamination. Bacterial infections commonly occur during surgical interventions, which could negatively impact tissue growth, delay wound recovery, increase risks of complications and even cause death [54,55,56]. *S. aureus* is a pathogen regarded as one of the most common sources of hospital-acquired infections and the main cause of surgical-wound infection [57]. In the US alone, nearly 500,000 hospitalized patients suffer from *S. aureus* infections or its antibiotic-resistant strains, such as methicillin-resistant *S. aureus* (MRSA), and death rate of the infection is around 10% [57]. Therefore, scaffolds with intrinsic antimicrobial activity could be beneficial during surgical implantations of tissue-engineered scaffolds, as they could reduce risks of infection. Decomposition of CP from the composite scaffolds in aqueous media produces calcium hydroxide and hydrogen peroxide, which is known to exhibit a potent antimicrobial effect on *S. aureus* [58,59]. Our antibacterial study demonstrated that the concentration of CP should be high enough (>2.5%) to have an inhibitory action against *S. aureus*, while remaining cytocompatible.

Since the main goal of developing oxygen-releasing scaffolds is to provide a better environment for cell metabolism, we evaluated the metabolic activity of patient-derived BM-MSCs. Our results suggest that oxygen release improved cell survival and growth by mitigating the local hypoxic environment. We also observed that cell viability is even higher when catalase was used, as the enzyme depletes hydrogen peroxide [46]. Overall, the composite scaffold could temporarily supply oxygen prior to neovascularization. The engineered oxygen-releasing biomaterials were designed to ensure high metabolic activity of cells, prevent potential microbial infections and degrade in a controlled fashion. The features make the composite scaffolds an attractive choice for a wide range of soft-tissue-engineering applications, such as wound healing, muscle regeneration and arterial prosthesis.

In order to translate the current platform from bench to bedside, a number of challenges still need to be addressed. Although oxygen release is sustained for a couple of days in the current strategy, initial burst release might be too high and potentially toxic to other cell types. To overcome this challenge, oxygen-generating scaffolds could be coated with an additional hydrophilic polymer layer, using coaxial electrospinning. Although we investigated the effect of oxygen release on cell growth and metabolic activity, further investigations are required to better understand the influence of oxygen release on cell migration and differentiation, and ultimately tissue growth in vivo. Finally, preclinical studies to assess long-term biocompatibility and degradation of the composite scaffolds are critical, which in general is an important factor for successful application of biomaterials for tissue-engineering applications.

## 5. Conclusions

The oxygen-releasing scaffolds composed of PGS/PCL polymer blends and CP nanoparticles were successfully fabricated by combining high-energy ball-milling and electrospinning techniques. The morphological structure, composition and coherency of the composite scaffold were confirmed via various characterization techniques. By adjusting the concentration of CP, the composite scaffold was engineered to supply oxygen for several days, meant to overcome oxygen deprivation prior to neovascularization of implanted tissues. The release of oxygen from the scaffold significantly improved the cell metabolic activity by alleviating a hypoxic environment of the cells cultured on the scaffolds. Moreover, by-products of CP were found to be effective for inhibiting bacterial pathogens associated with surgical infections. Finally, other features of the composite scaffolds related to tissue engineering, such as degradation profile, protein adsorption capacity can be adjusted by changing the composition of the scaffolds. Collectively, these results suggest that the developed composite scaffolds have great potential for various tissue-engineering applications involving ischemic conditions, including in chronic wound dressing and tissue engineering.

## Figures and Tables

**Table 1:** Abbreviations and compositions of prepared scaffolds.

Sample Name	PGS (% *wt./v*)	PCL (% *wt./v*)	CaO_2_ (% *wt./v*)	Catalase (mg/mL)
CP	0	0	100	-
CP0	10	10	0	-
CP1	10	10	1	-
CP2.5	10	10	2.5	-
CP5	10	10	5	-
CP10	10	10	10	-
CP2.5C	10	10	2.5	1
CP5C	10	10	5	1

**Table 2:** Atomic percentage for each element in the samples identified by EDX.

Sample Name	Carbon (%)	Oxygen (%)	Calcium (%)
CP	8.19	68.05	23.76
CP0	76.78	23.22	0
CP1	75.8	24.77	0.2
CP2.5	70.39	28.95	0.66
CP5	69.35	29.09	1.59
CP10	62.02	34.7	3.28

## Supplementary Materials

The following are available online at https://www.mdpi.com/2073-4360/12/6/1233/s1. Figure S1: EDX spectra of the composite scaffold before (a) and after (b) degradation in PBS buffer media. Figure S2: Protein adsorption capacity of the scaffolds, the data presented as amount of adsorbed protein in each milligram of loaded scaffolds.

## References

[1] Slaughter B.V., Khurshid S.S., Fisher O.Z., Khademhosseini A., Peppas N.A. (2009). Hydrogels in Regenerative Medicine. Adv. Mater..

[2] Suvarnapathaki S., Wu X., Lantigua D., Nguyen M.A., Camci-Unal G. (2019). Breathing life into engineered tissues using oxygen-releasing biomaterials. NPG Asia Mater..

[3] Hasan A., Memic A., Annabi N., Hossain M., Paul A., Dokmeci M.R., Dehghani F., Khademhosseini A. (2013). Electrospun scaffolds for tissue engineering of vascular grafts. Acta Biomater..

[4] Barua S., Chattopadhyay P., Aidew L., Buragohain A.K., Karak N. (2014). Infection-resistant hyperbranched epoxy nanocomposite as a scaffold for skin tissue regeneration. Polym. Int..

[5] Willerth S.M., Sakiyama-Elbert S.E. (2019). Combining Stem Cells and Biomaterial Scaffolds for Constructing Tissues and Cell Delivery. StemJournal.

[6] Xiao Y., Ahadian S., Radisic M. (2017). Biochemical and Biophysical Cues in Matrix Design for Chronic and Diabetic Wound Treatment. Tissue Eng. Part B Rev..

[7] Camci-Unal G., Alemdar N., Annabi N., Khademhosseini A. (2013). Oxygen Releasing Biomaterials for Tissue Engineering. Polym. Int..

[8] Gholipourmalekabadi M., Zhao S., Harrison B.S., Mozafari M., Seifalian A. (2016). Oxygen-Generating Biomaterials: A New, Viable Paradigm for Tissue Engineering?. Trends Biotechnol..

[9] Sarker M., Chen X., Schreyer D. (2015). Experimental approaches to vascularisation within tissue engineering constructs. J. Biomater. Sci. Polym. Ed..

[10] Becquart P., Cambon-Binder A., Monfoulet L.-E., Bourguignon M., Vandamme K., Bensidhoum M., Petite H., Logeart-Avramoglou D. (2012). Ischemia Is the Prime but Not the Only Cause of Human Multipotent Stromal Cell Death in Tissue-Engineered Constructs In Vivo. Tissue Eng. Part A.

[11] LaVan F.B., Hunt T.K. (1990). Oxygen and wound healing. Clin. Plast. Surg..

[12] Shiekh P.A., Singh A., Kumar A. (2018). Oxygen-Releasing Antioxidant Cryogel Scaffolds with Sustained Oxygen Delivery for Tissue Engineering Applications. ACS Appl. Mater. Interfaces.

[13] Nomi M., Atala A., De Coppi P., Soker S. (2002). Principals of neovascularization for tissue engineering. Mol. Asp. Med..

[14] Derakhshandeh H., Aghabaglou F., McCarthy A., Mostafavi A., Wiseman C., Bonick Z., Ghanavati I., Harris S., Kreikemeier-Bower C., Basri S.M.M. (2020). A Wirelessly Controlled Smart Bandage with 3D-Printed Miniaturized Needle Arrays. Adv. Funct. Mater..

[15] Memic A., Abudula T., Mohammed H.S., Navare K.J., Colombani T., Bencherif S.A. (2019). Latest Progress in Electrospun Nanofibers for Wound Healing Applications. ACS Appl. Bio Mater..

[16] Abudula T., Saeed U., Memic A., Gauthaman K., Hussain M.A., Al-Turaif H. (2019). Electrospun cellulose Nano fibril reinforced PLA/PBS composite scaffold for vascular tissue engineering. J. Polym. Res..

[17] Rinoldi C., Fallahi A., Yazdi I.K., Paras J.C., Kijeńska-Gawrońska E., Santiago G.T.-D., Tuoheti A., Demarchi D., Annabi N., Khademhosseini A. (2019). Mechanical and Biochemical Stimulation of 3D Multilayered Scaffolds for Tendon Tissue Engineering. ACS Biomater. Sci. Eng..

[18] Khalili S., Khorasani S.N., Razavi S.M., Hashemibeni B., Tamayol A. (2018). Nanofibrous Scaffolds with Biomimetic Composition for Skin Regeneration. Appl. Biochem. Biotechnol..

[19] Khan F., Aldhahri M., Hussain M.A., Gauthaman K., Memic A., Abuzenadah A., Kumosani T., Barbour E., Alothmany N.S., Aldhaheri R. (2018). Encapsulation of 5-Flurouracil into PLGA Nanofibers and Enhanced Anticancer Effect in Combination with Ajwa-Dates-Extract (Phoenix dactylifera L.). J. Biomed. Nanotechnol..

[20] Saghazadeh S., Rinoldi C., Schot M., Kashaf S.S., Sharifi F., Jalilian E., Nuutila K., Giatsidis G., Mostafalu P., Derakhshandeh H. (2018). Drug delivery systems and materials for wound healing applications. Adv. Drug Deliv. Rev..

[21] Rinoldi C., Kijeńska-Gawrońska E., Chlanda A., Choińska E., Khenoussi N., Tamayol A., Khademhosseini A., Swieszkowski W. (2018). Nanobead-on-string composites for tendon tissue engineering. J. Mater. Chem. B.

[22] Abudula T., Saeed U., Salah N., Memic A., Al-Turaif H. (2018). Study of Electrospinning Parameters and Collection Methods on Size Distribution and Orientation of PLA/PBS Hybrid Fiber Using Digital Image Processing. J. Nanosci. Nanotechnol..

[23] Oh S.H., Ward C.L., Atala A., Yoo J.J., Harrison B.S. (2009). Oxygen generating scaffolds for enhancing engineered tissue survival. Biomaterials.

[24] Harrison B.S., Eberli D., Lee S.J., Atala A., Yoo J.J. (2007). Oxygen producing biomaterials for tissue regeneration. Biomaterials.

[25] Pedraza E., Coronel M.M., Fraker C.A., Ricordi C., Stabler C.L. (2012). Preventing hypoxia-induced cell death in beta cells and islets via hydrolytically activated, oxygen-generating biomaterials. Proc. Natl. Acad. Sci. USA.

[26] Fraker C., Mendez A.J., Stabler C.L. (2011). Complementary Methods for the Determination of Dissolved Oxygen Content in Perfluorocarbon Emulsions and Other Solutions. J. Phys. Chem. B.

[27] Northup A., Cassidy D.P. (2008). Calcium peroxide (CaO_2_) for use in modified Fenton chemistry. J. Hazard. Mater..

[28] Cassidy D.P., Irvine R.L. (1999). Use of calcium peroxide to provide oxygen for contaminant biodegradation in a saturated soil. J. Hazard. Mater..

[29] Wang J., Zhu Y., Bawa H.K., Ng G., Wu Y., Libera M., Van Der Mei H., Busscher H., Yu X. (2010). Oxygen-Generating Nanofiber Cell Scaffolds with Antimicrobial Properties. ACS Appl. Mater. Interfaces.

[30] Jeffries E.M., Allen R., Gao J., Pesce M., Wang Y. (2015). Highly elastic and suturable electrospun poly(glycerol sebacate) fibrous scaffolds. Acta Biomater..

[31] Sundback C.A., Shyu J.Y., Wang Y., Faquin W.C., Langer R.S., Vacanti J.P., Hadlock T.A. (2005). Biocompatibility analysis of poly(glycerol sebacate) as a nerve guide material. Biomaterials.

[32] Tamayol A., Najafabadi A.H., Mostafalu P., Yetisen A.K., Commotto M., Aldhahri M., Abdel-Wahab M.S., Najafabadi Z.I., Latifi S., Akbari M. (2017). Biodegradable elastic nanofibrous platforms with integrated flexible heaters for on-demand drug delivery. Sci. Rep..

[33] Memic A., Aldhahri M., Tamayol A., Mostafalu P., Abdel-Wahab M.S., Samandari M., Moghaddam K.M., Annabi N., Bencherif S.A., Khademhosseini A. (2017). Nanofibrous Silver-Coated Polymeric Scaffolds with Tunable Electrical Properties. Nanomaterials.

[34] Abudula T., Gzara L., Simonetti G., Alshahrie A., Salah N., Morganti P., Chianese A., Fallahi A., Tamayol A., Bencherif S.A. (2018). The Effect of Poly (Glycerol Sebacate) Incorporation within Hybrid Chitin–Lignin Sol–Gel Nanofibrous Scaffolds. Materials.

[35] Kalakonda P., Aldhahri M.A., Abdel-Wahab M.S., Tamayol A., Moghaddam K.M., Benrached F., Pain A., Khademhosseini A., Memic A., Chaieb S. (2017). Microfibrous silver-coated polymeric scaffolds with tunable mechanical properties. RSC Adv..

[36] Najafabadi A.H., Tamayol A., Annabi N., Ochoa M., Mostafalu P., Akbari M., Nikkhah M., Rahimi R., Dokmeci M.R., Sonkusale S. (2014). Biodegradable nanofibrous polymeric substrates for generating elastic and flexible electronics. Adv. Mater..

[37] Salah N., Habib S.S., Khan Z.H., Memic A., Azam A., Alarfaj E., Zahed N., Al-Hamedi S. (2011). High-energy ball milling technique for ZnO nanoparticles as antibacterial material. Int. J. Nanomed..

[38] Stojilovic N. (2012). Why Can’t We See Hydrogen in X-ray Photoelectron Spectroscopy?. J. Chem. Educ..

[39] Hasan A., Soliman S., El Hajj F., Tseng Y.-T., Yalcin H.C., Marei H. (2018). Fabrication and In Vitro Characterization of a Tissue Engineered PCL-PLLA Heart Valve. Sci. Rep..

[40] Salehi S., Bahners T., Gutmann J.S., Gao S., Mader E., Fuchsluger T.A. (2014). Characterization of structural, mechanical and nano-mechanical properties of electrospun PGS/PCL fibers. RSC Adv..

[41] Madan S., Wasewar K.L., Kumar C.R. (2017). Optimization of adsorptive removal of α-toluic acid by CaO_2_ nanoparticles using response surface methodology. Resour. Technol..

[42] Rez M.F.A., Binobaid A., Alghosen A., Mirza E.H., Alam J., Fouad H., Hashem M., Alsalman H., Almalak H.M., Mahmood A. (2017). Tubular poly (ε-caprolactone)/chitosan nanofibrous scaffold prepared by electrospinning for vascular tissue engineering applications. J. Biomater. Tissue Eng..

[43] Corrêa A.C., Carmona V.B., Simão J.A., Mattoso L.H.C., Marconcini J.M. (2017). Biodegradable blends of urea plasticized thermoplastic starch (UTPS) and poly(ε-caprolactone) (PCL): Morphological, rheological, thermal and mechanical properties. Carbohydr. Polym..

[44] An Y., Wang S., Li R., Shi D., Gao Y., Song L. (2019). Effect of different nucleating agent on crystallization kinetics and morphology of polypropylene. Polymers.

[45] Liu W.J., Yang H., Wang Z., Dong L.S., Liu J.J. (2002). Effect of nucleating agents on the crystallization of poly(3-hydroxybutyrate-co-3-hydroxyvalerate). J. Appl. Polym. Sci..

[46] Symons M., Rusakiewicz S., Rees R., Ahmad S. (2001). Hydrogen peroxide: A potent cytotoxic agent effective in causing cellular damage and used in the possible treatment for certain tumours. Med. Hypotheses.

[47] Siqueira J.F., Lopes H.P. (1999). Mechanisms of antimicrobial activity of calcium hydroxide: A critical review. Int. Endod. J..

[48] Zhang H., Dalisson B., Tran S., E Barralet J. (2018). Preservation of Blood Vessels with an Oxygen Generating Composite. Adv. Health Mater..

[49] Crespy D., Friedemann K., Popa A.M. (2012). Colloid-Electrospinning: Fabrication of Multicompartment Nanofibers by the Electrospinning of Organic or/and Inorganic Dispersions and Emulsions. Macromol. Rapid Commun..

[50] Yin C.-G., Ma Y., Liu Z.-J., Fan J.-C., Shi P., Xu Q.-J., Min Y.-L. (2019). Multifunctional boron nitride nanosheet/polymer composite nanofiber membranes. Polymer.

[51] Wu X., Stroll S.I., Lantigua D., Suvarnapathaki S., Camci-Unal G. (2019). Eggshell particle-reinforced hydrogels for bone tissue engineering: An orthogonal approach. Biomater. Sci..

[52] Mao D., Li Q., Li D., Chen Y., Chen X., Xu X. (2018). Fabrication of 3D porous poly(lactic acid)-based composite scaffolds with tunable biodegradation for bone tissue engineering. Mater. Des..

[53] Zhang K., Zhou Y., Xiao C., Zhao W., Wu H., Tang J., Li Z., Yu S., Li X., Min L. (2019). Application of hydroxyapatite nanoparticles in tumor-associated bone segmental defect. Sci. Adv..

[54] Dai T., Wang C., Wang Y., Xu W., Hu J., Cheng Y. (2018). A Nanocomposite Hydrogel with Potent and Broad-Spectrum Antibacterial Activity. ACS Appl. Mater. Interfaces.

[55] Kazemzadeh-Narbat M., Lai B.F., Ding C., Kizhakkedathu J.N., Hancock R.E., Wang R. (2013). Multilayered coating on titanium for controlled release of antimicrobial peptides for the prevention of implant-associated infections. Biomaterials.

[56] Li P., Poon Y.F., Li W., Zhu H.-Y., Yeap S.H., Cao Y., Qi X., Zhou C., Lamrani M., Beuerman R.W. (2010). A polycationic antimicrobial and biocompatible hydrogel with microbe membrane suctioning ability. Nat. Mater..

[57] Schlecht L.M., Peters B.M., Krom B.P., Freiberg J.A., Hänsch G.M., Filler S.G., Jabra-Rizk M.A., Shirtliff M. (2015). Systemic Staphylococcus aureus infection mediated by Candida albicans hyphal invasion of mucosal tissue. Microbiology.

[58] Estrela C., Pimenta F.C., Ito I.Y., Bammann L.L. (1999). Antimicrobial evaluation of calcium hydroxide in infected dentinal tubules. J. Endod..

[59] Estrela C., Pimenta F.C., Ito I.Y., Bammann L.L. (1998). In vitro determination of direct antimicrobial effect of calcium hydroxide. J. Endod..

